# MutL homologs in restriction-modification systems and the origin of eukaryotic MORC ATPases

**DOI:** 10.1186/1745-6150-3-8

**Published:** 2008-03-17

**Authors:** Lakshminarayan M Iyer, Saraswathi Abhiman, L Aravind

**Affiliations:** 1 National Center for Biotechnology Information, National Library of Medicine, National Institutes of Health, Bethesda, MD 20894, USA

## Abstract

The provenance and biochemical roles of eukaryotic MORC proteins have remained poorly understood since the discovery of their prototype MORC1, which is required for meiotic nuclear division in animals. The MORC family contains a combination of a gyrase, histidine kinase, and MutL (GHKL) and S5 domains that together constitute a catalytically active ATPase module. We identify the prokaryotic MORCs and establish that the MORC family belongs to a larger radiation of several families of GHKL proteins (paraMORCs) in prokaryotes. Using contextual information from conserved gene neighborhoods we show that these proteins primarily function in restriction-modification systems, in conjunction with diverse superfamily II DNA helicases and endonucleases. The common ancestor of these GHKL proteins, MutL and topoisomerase ATPase modules appears to have catalyzed structural reorganization of protein complexes and concomitant DNA-superstructure manipulations along with fused or standalone nuclease domains. Furthermore, contextual associations of the prokaryotic MORCs and their relatives suggest that their eukaryotic counterparts are likely to carry out chromatin remodeling by DNA superstructure manipulation in response to epigenetic signals such as histone and DNA methylation.

This article was reviewed by Arcady Mushegian and Gaspar Jekely.

## Introduction

The microchidia gene product (MORC1) was found to be required for the completion of prophase I of meiosis during mammalian spermatogenesis [1]. Disruption of the microrchidia gene also resulted in altered localization of the meiotic endonuclease, Spo11 [2]. It was noticed that MORC1 was the prototype of a novel family (MORC family) of eukaryotic chromatin proteins which possess a gyrase, histidine kinase, and MutL (GHKL) domain [3-5]. Phyletic analysis showed that the MORC proteins are widely distributed in eukaryotes, being present in most major crown group lineages (except fungi), apicomplexans and heteroloboseans, suggesting a relatively early origin in eukaryotes [5]. Consistent with a chromatin-associated role, the MORCs display fusions to several DNA- and peptide-binding domains, which are commonly found other eukaryotic chromatin proteins [5]. However, their exact functions in chromatin dynamics are poorly understood. The evolutionary origin of the MORC family also remains unclear, as their specific relationship to other conserved eukaryotic members of the GHKL superfamily remain unstudied.

### Identification of prokaryotic Restriction Modification systems encoding MORC family proteins

**Figure 1:**
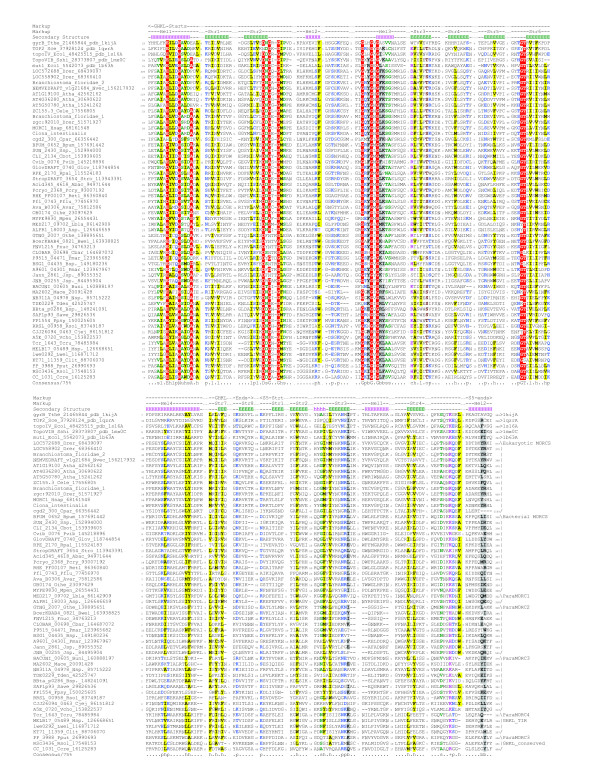
**Multiple alignment of the MORCs, MGHKls and some characterized GHKL domains**. Jpred predicted Secondary structure is shown above the alignment. The 75% consensus shown below the alignment was derived using the following classes of amino acids: hydrophobic (h: ALICVMYFW, yellow shading); the aliphatic subset of the hydrophobic class (l: LIV, yellow shading); hydrophobic (h: ACFILMVWY, yellow shading); small (s: ACDGNPSTV, green); the tiny subset of the small class (u: GAS, green); polar (p: CDEHKNQRST, blue); alcohol subset of polar (o: ST, brown); charged subset of polar (c: DEHKR, pink); positive subset of polar (+: HKR, pink). Amino acids in red background are completely conserved. Different subgroups of MORCS are indicated at the right end of the alignment. The sequences are denoted by their gene name followed by species abbreviation and GenBank Identifier separated by underscores. The species abbreviations are: *Abac: Acidobacteria bacterium; Atha: Arabidopsis thaliana; Avar: Anabaena variabilis; Bpum: Bacillus pumilus; Bsp.: Bacillus sp.; Bsp.: Bradyrhizobium sp.; Btau: Bos taurus; Buni: Bacteroides uniformis; Bwei: Bacillus weihenstephanensis; Cbar: Clostridium bartlettii; Cbot: Clostridium botulinum; Ccre: Caulobacter crescentus; Cele: Caenorhabditis elegans; Cjej: Campylobacter jejuni; Clit: Congregibacter litoralis; Cpar: Cryptosporidium parvum; Drer: Danio rerio; Fnuc: Fusobacterium nucleatum; Fpsy: Flavobacterium psychrophilum; Glov: Geobacter lovleyi; Gthe: Geobacillus thermodenitrificans; Hsap: Homo sapiens; Jsp.: Janibacter sp.; Jsp.: Jannaschia sp.; Lbla: Leeuwenhoekiella blandensis; Lwel: Listeria welshimeri; Mace: Methanosarcina acetivorans; Mpen: Mycoplasma penetrans; Msp.: Marinobacter sp.; Nsp.: Nitrobacter sp.; Nvec: Nematostella vectensis; Oihe: Oceanobacillus iheyensis; Pcry: Psychrobacter cryohalolentis; Pflu: Pseudomonas fluorescens; Pmar: Prochlorococcus marinus; Pput: Pseudomonas putida; Pvib: Prosthecochloris vibrioformis; Retl: Rhizobium etli; Rpal: Rhodopseudomonas palustris; Rsol: Ralstonia solanacearum; Save: Streptomyces avermitilis; Ssp.: Sulfurovum sp.; Stro: Salinispora tropica; Tcru: Thiomicrospira crunogena; Tden: Treponema denticola; Vcho: Vibrio cholerae*

To unravel the provenance and relationships of the MORC family, we initiated sequence profile searches using the PSI-BLAST program seeded with eukaryotic MORC proteins. These searches additionally retrieved several prokaryotic homologs (within first 3 iterations; e-value < .01), prior to other members of the GHKL superfamily. A multiple alignment of the eukaryotic MORCs with these prokaryotic homologs showed that both versions contained all four conserved motifs required for adenosine and phosphate binding in the GHKL superfamily (Fig. 1). Additionally, the prokaryotic homologs contained a C-terminal α+β domain with the same secondary structure as the S5-fold domain that is also found in a subset of the GHKL superfamily, namely the topoisomerase ATPase domains, MutL and Hsp90. This S5 domain usually provides a conserved basic residue, which might function similar to the arginine or lysine finger observed in various phosphohydrolase reactions [6]. These findings suggest that the MORCs are active enzymes capable of ATP hydrolysis. The prokaryotic and eukaryotic MORC homologs were unified to the exclusion of the other GHKL proteins by a specific GhXhhpXXRhl motif (h: hydrophobic, p: polar, X: any) in the S5-fold domain (Fig. 1). The prokaryotic MORC proteins were widely, but sporadically, distributed in several distantly related bacteria such as proteobacteria, cyanobacteria and actinobacteria, as well as certain crenarchaea and euryarchaea [7]. Additionally, in a phylogenetic tree versions from the same bacterial lineage (e.g. proteobacteria) often do not group together. This is suggestive of extensive lateral transfer of these genes between diverse prokaryotes.

**Figure 2:**
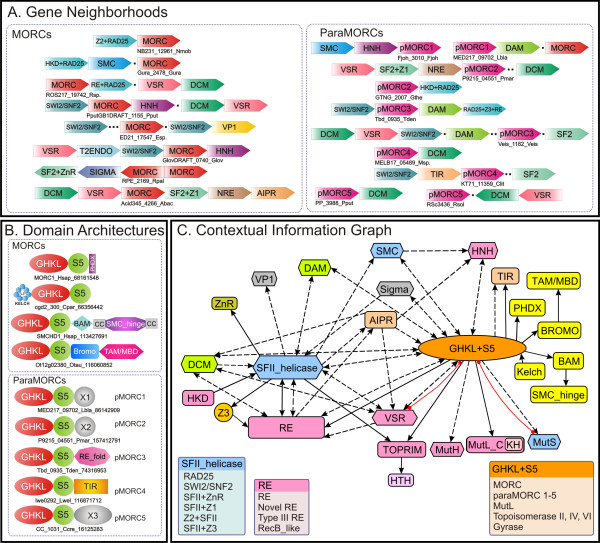
**Gene neighborhoods, domain architectures and contextual information graph of MORCS and related GHKL families**. A) The gene neighborhoods are shown for selected MORCs (left panel) and paraMORCS (right panel). The direction of arrows indicates transcriptional direction and dots indicate intervening regions that might contain additional genes. Representative gene names are shown below each operon type. B) Representative domain architectures of MORCs and related proteins are shown. SMC_hinge: Structural maintenance of chromosome protein hinge domain; HTH: helix-turn-helix domain; BAM: Bromo-associated motif (also known as bromo-associated homology domain); TAM/MBD: C-methyl-DNA-binding domain; Z1, Z2, Z3 and X1, X2, X3 are uncharacterized domains. C) Ordered graph showing contextual information from gene neighborhoods and domain architectures. Solid arrows indicated information from domain architectures and dotted lines indicate information from gene neighborhoods. The direction of arrows denotes the order of genes in operons or order of domains in proteins from N-terminal to C-terminal. Red edges correspond to physical interactions between the domains. Genes are shown with hexagons and domains are shown as rounded rectangles. The domain coloring highlights the major contextual themes that are consistently seen with these proteins even if the actual domains belong to different families or are structurally distinct. For example, domains occurring only in eukaryotes are in yellow, nucleases are colored pink, methylases in green and non-GHKL ATPases in blue. Standard gene names are used. Additional Gene name abbreviations include: DAM: DNA Adenine Methylase; DCM: DNA Cytosine Methylase; HKD: possible nuclease domain of Phospholipase D fold; NRE: Novel Restriction Endonuclease; T2ENDO: Type II Endonuclease; pMORC: paraMORC; AIPR: Abortive infection phage resistance protein, a protein commonly encoded by numerous RM operons. Species abbrevations are as in Figure 1. Additional abbreviations are as follows: *CPel: Candidatus Pelagibacter; Esp.: Erythrobacter sp.; Fjoh: Flavobacterium johnsoniae; Gura: Geobacter uraniireducens; Nmob: Nitrococcus mobilis; Otau: Ostreococcus tauri; Rsp.: Roseovarius sp.; Veis: Verminephrobacter eiseniae*.

To acquire functional insights on prokaryotic MORCs by means of contextual information, we systematically explored their conserved gene neighborhoods or predicted operons. Consequently, we uncovered several types of predicted operons encoding MORC proteins (Fig. 2). A consistent theme in the majority of these predicted gene-neighborhoods (~70%) was the presence of a gene encoding a superfamily II (SFII) helicase. In computational experiments, the probability that such an association occurs by chance alone in phylogenetically distinct bacterial lineages is less than .0001, suggesting that the MORC-SFII gene neighborhood was a significant functional association. Based on the evolutionary relationships of the SFII helicase we identified three major types of predicted operons (Fig. 2): 1) The first set of these encoded Rad25-like helicases, which are usually found in type III restriction-modification (RM) systems. Analysis of the Rad25-like helicases showed that they were commonly fused to C-terminal or N-terminal RE fold DNAses, as is typical of previously characterized type-III RM systems [8]. In a few instances, rather than a RE fold nuclease, we found an N-terminal HKD superfamily phosphoesterase domain, that could potentially function as a nuclease [9]. 2) The SFII helicase in the second group of predicted operons belonged to the SWI2/SNF2 family. These operons also often encoded DNAses belonging to the HNH (EndoVII fold) superfamily or EcoRII-like nucleases. Some of these neighborhoods also encoded a protein with a VP1/RAV DNA-binding domain, which has also been found in association with REs like EcoRII [10]. 3) The third type of operon encoded a distinct SFII helicase with a conserved C-terminal globular α+β domain possessing a DThhQXuRhFG motif (h: hydrophobic, u: tiny). These gene neighborhoods consistently contained an additional gene encoding an uncharacterized conserved protein. Sequence profile searches with these protein recovered the members of the RE fold and they displayed the E-D-ExK motifs typical of the classical RE superfamily [11].

The above gene neighborhoods also frequently contained genes for DNA methylases (mostly cytosine and rarely adenine) and predicted endonucleases of the very short patch repair (VSR) superfamily of the RE fold [11]. A minority of conserved gene neighborhoods combined MORC genes with those coding DNA methylases or REs, but lacked helicase genes. A subset of these of gene neighborhoods also encoded another distinct version of the GHKL superfamily (hereafter termed paraMORC1 family), with intact catalytic motifs and a distinct conserved C-terminal extension beyond the predicted S5 domain. We noticed that members of the paraMORC1 family might also co-occur in predicted operons with HNH endonucleases (Fig. 2). Thus, the predicted operons with prokaryotic MORC genes appear to predominantly encode a diverse set of RM systems.

### Related RM systems contain other distinct families of GHKL proteins

In course of the above analysis, we noticed some conserved gene-neighborhoods those were closely related to the above-described MORC-containing operons, but differed from them in lacking a MORC gene. Given that there are several instances of displacement of genes in operons by other genes coding functionally equivalent, but evolutionarily distinct proteins families, we examined these operons for potential replacements for the MORCs. To do this we systematically explored the related operons without MORCs and represented the neighborhood associations as a network diagram (Fig. 2). As result we discovered candidate genes that showed comparable neighborhood associations as the MORCs with the distinct SFII helicase genes, as well as DNA methylases, Vsr superfamily endonucleases and the above-described predicted endonucleases of the RE fold (Fig. 2). Strikingly, further sequence analysis of these candidates showed that they belonged to four new families of GHKL ATPases – paraMORC2-5. While being distantly related to the MORCs, and lacking the specific motif that unifies the MORC family, paraMORC1 and the newly identified families paraMORC2-5 showed a general architectural similarity to the MORCs. All of them possess N-terminal GHKL domains with conserved ATP-binding motifs combined to a C-terminal S5 domain. Of these, the paraMORC2 and paraMORC3 families are, like the MORCs, widely but sporadically distributed in distantly related bacteria and archaea, suggesting extensive lateral mobility. The paraMORC2 family is predominantly present in cyanobacteria and Gram-positive bacteria, whereas the paraMORC3 family is prevalent in proteobacteria and the bacteroidetes clade. The remaining two families (paraMORC4-5) are more infrequent, and patchy in their distributions.

Members of the paraMORC2 possess a strongly conserved ExxxH motif in the C-terminal part of their S5 domain, which might form a distinctive substrate interaction site of this family. The paraMORC3 family is typified by the presence of an additional conserved C-terminal globular domain. Sequence profile analysis of this conserved domain recovered significant hits to several RecB family nucleases and revealed the presence of a conserved E-D-ExK motifs [11], indicating that it is likely to be an endonuclease domain of the RE fold. Similarly, several members of the paraMORC4 and paraMORC5 families also possessed their own conserved C-terminal extensions. Analysis of the extension of the paraMORC4 family showed that it is a TIR domain [12], which might alternatively also be encoded in a standalone form by a neighboring gene. The TIR domain possesses a Rossmannoid fold, with a potential ligand-binding site in the classical position of this fold, formed by conserved polar residues in the loop between the first strand and helix and the region immediately C-terminal to the 3^rd ^conserved strand (usually a Sx [ND] motif) [12]. In the TIR domains associated with the paraMORC4 family and their close relatives a highly conserved Hx [ST]xD motif is present in the loop between first strand and helix. This could potentially form a catalytic or ligand-binding site of these domains. Given the presence of similarly linked C-terminal nuclease domains in paraMORC3 and other GHKL superfamily members like MutL and topoisomerases (see below) it is possible that this version of the TIR domain is a nuclease. Alternatively, it might function as an allosteric nucleotide or nucleic acid binding site. The C-terminal domain of the paraMORC5 family appears unrelated to any other previously known domain, but contains some highly conserved polar residues (e.g a HxH motif), which could again define an uncharacterized nuclease active site.

Thus, in conclusion the paraMORC1-5 families appear to define a group of GHKL proteins that are likely to possess similar functions as the MORCs, predominantly as components of RM systems. However, a subset of the paraMORC3 family (i.e. those with C-terminal endonuclease domains), mainly in proteobacteria and bacteriodetes occur as standalone genes without any conserved neighborhood associations. This might imply that these members of the paraMORC3 family might have acquired distinct functions independent of the RM systems or in a few species may function with RM systems at unlinked locations.

### Mechanistic and evolutionary implications of the contextual associations of the MORCs and paraMORCs

The above-described architectural and contextual observations on MORCs and paraMORCs provide considerable clues regarding their potential functional mechanisms. Their contextual associations imply direct physical and functional associations between these ATPases, one or more distinct DNAses and SFII helicases in these RM systems. These are highly reminiscent of the architectures and functional associations of other members of the GHKL superfamily with DNA-related functions. In both topoisomerases and MutL the GHKL domain functionally interacts with a nuclease domain that is either present C-terminal to the ATPase domain or as a standalone polypeptide. In the case of topoisomerases the nuclease is a TOPRIM domain [13]. In the case of MutL from *Escherichia coli *and several related proteobacteria, the nuclease in mismatch repair (MMR) is a standalone protein MutH, while in very short patch pair it is the vsr endonuclease; both nucleases contain a RE fold [11,14]. In a subset of the eukaryotic Mlh proteins (e.g. PMS2) and their orthologs from the majority of bacteria, the nuclease activity is in the same polypeptide, supplied by a module C-terminal to the S5 domain [15]. This module has a metal-binding nuclease catalytic site in a unique 4-stranded α+β domain into which a KH domain is inserted after the 3^rd ^strand to form a composite nucleic acid interacting surface. Topoisomerase activity is often combined with the action of DNA helicases, and in some cases like the reverse gyrase the two modules are combined into a single polypeptide. In bacteria like *E. coli*, MutL physically interacts with the DNA helicase UvrD which unwinds DNA starting from the site of nick to initiate degradation of the strand with the mismatch [16]. In the case of bacterial MMR and VSR the discrimination of the correct template strand is brought about by means of an epigenetic mark of cytosine hemimethylation [17,18]. This functional association is again reminiscent of the association of the MORCs and paraMORCs with methyltransferases (including those mediating hemimethylation like Type III RM systems [8]).

In mechanistic terms, the GHKL+S5 modules of both topoisomerases and MutLs constitute engines that use ATP hydrolysis to drive alterations in protein-protein interactions in the complexes they are part of, as well as mediate large movements of DNA strands – topoisomerization and looping of DNA between the epigenetic marks and mismatch sites [17]. Based on these models we propose that the MORCs and the paraMORCs in the RM operons are similarly involved in DNA looping, probably linking the site of endonucleolytic cleavage with the recognition site of the restriction enzyme. Associated DNA helicases probably contribute to the unwinding of the DNA starting at the sites of the nicks. This mechanistic interpretation of the prokaryotic MORCs and paraMORCs also furnishes functional predictions regarding the eukaryotic MORCs. Firstly, the presence of a distinct ancient eukaryotic lineage with a fusion of the MORC GHKL+S5 module to hinge and coiled-coil domains also found in SMC-like ATPases suggests that they might function as analogous chromosome looping enzymes [5]. Secondly, the genetic link to the meiotic endonuclease Spo11, the archaeal Topo VI ortholog, suggests that at least MORC1 might function in combination with this protein in DNA strand manipulation during meiotic recombination [2]. Several of the domains linked to the MORC module in eukaryotic polypeptides, such as the Bromo, Bromo-associated motif (BAM), PHDX/ZfCW and the TAM/MBD are known or predicted to bind potential epigenetic markers in chromatin such as modified histone tails and methylated DNA [5,19]. Thus, like their prokaryotic equivalents the eukaryotic MORCs might respond to these epigenetic signals to catalyze alterations in chromosome superstructure.

In evolutionary terms, the association between nucleases and the GHKL+S5 module is an ancient one with at least two representatives in the topoisomerases traceable to the last universal common ancestor. MutL in contrast appears to be bacterial innovation. Given the similarities between the MORCs and paraMORCs from RM systems on one hand and MutL in MMR and VSR on the other, it appears likely that these systems arose early in bacterial evolution from an ancestral version that functionally united GHKL ATPases, nucleases and helicases. The latter seems to have acquired a key DNA repair role early in bacterial evolution, in conjunction with the mismatch recognizing ABC ATPase MutS, resulting in them being fixed in the bacterial superkingdom. The former group like all RM systems was highly laterally mobile. This is also supported by a survey of their chromosomal locations, which suggests that they behave like transposable elements integrating in "hotspots" that contain other mobile elements [Additional file 1]. However, on a few occasions they might have been recruited for some form of DNA mismatch repair as suggested by the versions which occur in non-RM contexts. This can be compared to the recruitment of the restriction enzyme MutH to the MMR system in proteobacteria with the concomitant degeneration of the endogenous nuclease of MutL. Finally, the greater diversity of these RM system-associated MORCs and paraMORCs in bacteria suggests that eukaryotes acquired them in a single lateral transfer event from a bacterial source. Following, this transfer, their ancestral properties in responding to epigenetic signals like DNA methylation appear to have been reused in contexts unique to the eukaryotic chromatin. Thus, MORCs join a growing group of eukaryotic chromatin-modifying enzymes, such as DNA methylases, SWI/SNF ATPases and the HIRAN domain that might have emerged from RM systems and other comparable mobile DNA elements of bacteria [5,20].

## Materials and methods

Gene neighborhoods were determined using a custom script that uses completely sequenced genomes or whole genome shot gun sequences to derive a table of gene neighbors centered on a query gene. Then the BLASTCLUST program is used to cluster the products in the neighborhood and establish conserved co-occurring genes. These conserved gene neighborhood are then sorted as per a ranking scheme based on occurrence in at least one other phylogenetically distinct lineage ("phylum" in NCBI Taxonomy database), complete conservation in a particular lineage ("phylum") and physical closeness on the chromosome indicating sharing of regulatory -10 and -35 elements. For obtaining an approximate probability of the neighborhood associations we chose 11 phylogenetically distinct genomes that contained MORC proteins and reconstituted 10,000 replicates of "pseudo-genomes" of the same size by shuffling their combined gene-pool. We introduced the constraint that each such genome possessed at least 1 MORC and 10 SFII helicases. The probability of association between MORCs and other genes in the neighborhoods by chance was derived from the observed co-occurrence in the pseudo-genome replicates. Profile searches were conducted using the PSI-BLAST program with either a single sequence or an alignment used as the query, with a default profile inclusion expectation (E) value threshold of 0.01 [21]. Multiple alignments were constructed using the PCMA and Kalign programs followed by manual adjustments based on PSI-BLAST results. Protein secondary structure was predicted using a multiple alignment as the input for the JPRED program. Phylogenetic trees were constructed with the MEGA4 package [22].

## Competing interests

The author(s) declare that they have no competing interests.

## Reviewer's comments

Reviewer 1: Arcady Mushegian

*1. Par. 2: "This is suggestive of extensive lateral transfer of these genes between diverse prokaryotes." This sporadic distribution is equally suggestive of extensive gene loss in many lineages. In order to argue for lateral transfer in earnest, either make a quantitative argument that involves the number of branches, their topology, and gene gain-to-loss ratio, or present case studies of gene tree and species tree incongruency (the same type of statement is at the end of par.6, the same criticism). In the concluding paragraph, there are some auxiliary considerations lending support to the LGT scenario, but they perhaps need to be brought up earlier*.

### Authors' response

A phylogenetic tree of the MORC ATPases is provided as supplementary material [7]. The tree recovers the various MORC families defined in the text. Further, within each family, the protein tree is very different from the bacterial species tree (i.e. the predominant phylogenetic signal in the bacterial genomes). This is even seen in the case of MORC proteins from closely related species. For example, within the classical bacterial MORCs, the proteobacterial homologs do not specifically cluster together to the exclusion of other bacterial groups. Thus extensive lateral transfer between diverse prokaryotic species is the best explanation of this data.

2. *Par. 3–5: "Consequently, we uncovered several types of predicted operons encoding MORC proteins *(Fig. 2). *A consistent theme in majority of these predicted gene-neighborhoods was the presence of a gene encoding a superfamily II (SFII) helicase." – it would not hurt to make this and similar following statements more quantitative. What is a definition of the neighborhood? Most prokaryotes have perhaps at least 10–15 helicases; with roughly half of them being SFII and taking the "neighborhood" size to be 10 genes, we can estimate that perhaps 3% of all genes have an SFII helicase nearby. How many MORCs have a helicase in the neighborhood, how many do not have one? Is the probability of a MORC having a helicase nearby higher than that of a random gene (and is perhaps a probability of not having one lower than of a random gene)? Helicase/nuclease tandems and fusions are common in all genomes – are nucleases in the same neighborhood more likely to be found than would be predicted solely by their association with helicases? Materials and methods hint at some sort of justification, i.e. the use of conserved orthologous pairs, but there is no reference to BLASTCLUST nor explaination of what is measured by the program*.

### Authors' response

We do not dispute the estimate of the percentage of genes of having a SF-II helicase in the vicinity, but the available evidence suggests that the associations reported here are not artefacts of chance association followed by retention due to phylogenetic closeness. We evaluated the probability of the associations of MORC and SFII proteins by a computational experiment of generating "pseudo-genomes". We chose 11 phylogenetically distinct genomes that contained MORC proteins and reconstituted 10,000 replicates of "pseudo-genomes" of the same size by shuffling their combined gene-pool. We introduced the constraint that each such genome possessed at least 1 MORC and 10 SFII helicases (based on real mean number of detected SFIIs). The probability of association between MORCs and other genes in the neighborhoods by chance was derived from the observed co-occurrence in the pseudo-genome replicates. The probability of observing a MORC next to *any *SFII in at least 7 of the genomes (~70% of genomes in the real data show the MORC-SFII linkage) was <.0001. This probability reduces further if introduce the realistic constraint that the SFII helicases should belong to one of the 3 observed families.

3. *Par. 6 and *Fig. 2C: *Is the network shown on *Fig. 2C* a union of all interactions/relationships inferred for individual MORC proteins and SFII helicases that co-occur with them? In other words, does any given MORC or paraMORC have relationships to only a small subset of all other proteins in the chart? If the answer is yes, perhaps it is worth explaining what the chart is supposed to convey*.

### Authors' response

The contextual information graph reflects the union of all interactions between members of the GHKL family of proteins that are fused to an S5 (a subset of which include the MORCs) and other domains/proteins. The graph highlights the major contextual themes that are consistently seen with these proteins even if the actual domains belong to different sequence or structural families. Thus, distinct chromatin associated domains are colored yellow, diverse nucleases in pink and distinct ATPases in blue. (See figure legend).

4. *Par. 8. "This might imply that these members of the paraMORC3 family might have acquired distinct functions independent of the RM systems." – Does this imply it or not? Do those species that have a standalone versions of paraMORC3 also have (unlinked) orthologs of the R-M genes that are linked in other species? If the answer is yes, then the neighborhood may be split, but the system is still there, perhaps even co-regulated?*

### Authors' response

In principle, it is definitely possible and perhaps true for a small set of the species that these paraMORC3s function with an RM system, that is co-regulated and at an unlinked location in the genome. However, for most of these species, we failed to detect any solo R-M systems like those associated with other MORC families in these species thereby leading us to speculate about the evolution of a distinct function. We have changed the sentence to accommodate the former possibility.

Reviewer #2: Gaspar Jekely

1. *Introduction "being present in most major crown group lineages". The use of the term crown group is not fortunate in this sense (i.e. excluding apicomplexans etc. that are also descendants of the last common eukaryotic ancestor = crown group*).

### Authors' response

We agree that with the above phylogenetic definition of the term crown group we would be excluding many eukaryotes. However, crown group has also been defined as: "All the taxa descended from a major cladogenesis event, recognized by possessing the clade's synapomorphy" (see ). Several phylogenetic analyses strongly support the monophyly of animals, plants, fungi and amoebozoans with respect to the other eukaryotes. Hence we use the term in the sense mentioned above for this group of lineages. We used this term to emphasize the loss of the MORC proteins in the fungi.

2. *The authors propose that the eukaryotic MORC family came from eubacteria and not archaebacteria where MORCs are also present. I think diversity alone is not too strong support for this*.

### Authors' response

We provide a phylogenetic analysis of the MORCs and related ATPases in the supplementary material. In our survey we only detected three archaeal MORC proteins. Further, the phylogenetic tree shows that the archaeal versions are not specifically closer to the eukaryotic MORCs, but instead group with different bacterial versions. This is suggestive of lateral acquisition of the archaeal and eukaryotic MORCs from bacterial homologs.

## Supplementary Material

Additional file 1 Supplementary information of MORCs. The complete list of conserved neighborhoods, architectures, alignments and phylogenetic tree of various domains discussed in this article, and references for the sequence analysis methods are provided in additional file 1. They can also be accessed from: Click here for file
