# Supplementary material for: MutL homologs in restriction-modification systems and the origin of eukaryotic MORC ATPases
Source: Biol Direct. 2008 Mar 17;3:8. doi: 10.1186/1745-6150-3-8 (PMC2292703; doi:10.1186/1745-6150-3-8)
Supplement: Additional file 1 — Supplementary information of MORCs. The complete list of conserved neighborhoods, architectures, alignments and phylogenetic tree of various domains discussed in this article, and references for the sequence analysis methods are provided in additional file 1. They can also be accessed from: [file 1745-6150-3-8-S1.html]

SUPPLEMENTARY MATERIAL FOR: The origin of eukaryotic MORC ATPases from prokaryotic restriction-modification systems and recruitment of MutL-like ATPases in DNA diverse manipulation systems
